# The correlation between point-of-care ultrasound and digital tomosynthesis when used with suspected COVID-19 pneumonia patients in primary care

**DOI:** 10.1186/s13089-022-00257-7

**Published:** 2022-02-22

**Authors:** Pablo Fabuel Ortega, Noelia Almendros Lafuente, Sandra Cánovas García, Laura Martínez Gálvez, Aurora González-Vidal

**Affiliations:** 1 Vistalegre-La Flota Health Centre, Paseo Ing. Sebastián Feringán, 18, 30007 Murcia, Spain; 2 Mario Spreáfico-Archena Health Centre, Archena, Murcia Spain; 3 Department of Information and Communication Engineering Faculty of Computer Sciences, University of Murcia, Murcia, Spain

**Keywords:** Coronavirus infection, Ultrasonography, Family practice, Pneumonia, Radiography, Thoracic

## Abstract

**Background:**

The use of lung ultrasound (LU) with COVID-19 pneumonia patients should be validated in the field of primary care (PC). Our study aims to evaluate the correlation between LU and radiographic imaging in PC patients with suspected COVID-19 pneumonia.

**Methods:**

This observational, prospective and multicentre study was carried out with patients from a PC health area whose tests for COVID-19 and suspected pneumonia had been positive and who then underwent LU and a digital tomosynthesis (DT). Four PC physicians obtained data regarding the patients’ symptoms, examination, medical history and ultrasound data for 12 lung fields: the total amount of B lines (zero to four per field), the irregularity of the pleural line, subpleural consolidation, lung consolidation and pleural effusion. These data were subsequently correlated with the presence of pneumonia by means of DT, the need for hospital admission and a consultation in the hospital emergency department in the following 15 days.

**Results:**

The study was carried out between November 2020 and January 2021 with 70 patients (40 of whom had pneumonia, confirmed by means of DT). Those with pneumonia were older, had a higher proportion of arterial hypertension and lower oxygen saturation (sO_2_). The number of B lines was higher in patients with pneumonia (16.53 vs. 4.3, *p* < 0.001). The area under the curve for LU was 0.87 (95% CI 0.78–0.96, *p* < 0.001), and when establishing a cut-off point of six B lines or more, the sensitivity was 0.875 (95% CI 0.77–0.98, *p* < 0.05), the specificity was 0.833 (95% CI 0.692–0.975, *p* < 0.05), the positive-likelihood ratio was 5.25 (95% CI 2.34–11.79, *p* < 0.05) and the negative-likelihood ratio was 0.15 (95% CI 0.07–0.34, *p* < 0.05). An age of ≥ 55 and a higher number of B lines were associated with admission. Patients who required admission (*n* = 7) met at least one of the following criteria: ≥ 55 years of age, sO_2_ ≤ 95%, presence of at least one subpleural consolidation or ≥ 21 B lines.

**Conclusions:**

LU has great sensitivity and specificity for the diagnosis of COVID-19 pneumonia in PC. Clinical ultrasound findings, along with age and saturation, could, therefore, improve decision-making in this field.

## Introduction

During the recent SARS-CoV-2 (severe acute respiratory syndrome coronavirus 2) pandemic, lung ultrasound (LU) has been a useful tool in the diagnosis and management of COVID-19 pneumonia (coronavirus disease 2019) [1].

The low sensitivity of chest radiography has led to the proposal that computed tomography (CT) could be used as the gold standard [2]. However, the limited access to it, its risk of radiation and the saturation of diagnostic imaging services signifies that there is a growing need to carefully consider its indication [3] and to seek alternatives by which to identify the existence of lung damage and its severity.

Digital tomosynthesis (DT) and LU have, therefore, been proposed as means to improve the diagnostic precision of chest radiography [4–11]. DT is considered to be an emerging application that has some of the tomographic benefits of CT, since it provides multiple anatomic images, but has a lower cost and radiation dose [12]. It is normally used as a diagnostic tool for breast cancer, but has nevertheless been shown to be not only useful in lung pathologies, but also better than simple chest radiography in the case of pulmonary nodules or cystic fibrosis, as it reduces superposition artefacts and improves resolution [13, 14]. Given this, it could improve the diagnostic value of chest radiography with regard to assessing COVID-19 lung involvement [4]. With regard to LU, it has also proved to be useful and to have a good correlation with CT [6, 7] and to be superior to chest radiography [8]. However, although LU has been proposed as a means to optimise primary care (PC) patients’ well-being [9], most studies concerning its sensitivity and specificity have been carried out in a hospital setting [10, 11].

The objective of this study is to evaluate the correlation between LU and DT in PC patients with suspected COVID-19 pneumonia. A description of the demographic, clinical, ultrasound and radiological characteristics of patients with suspected COVID-19 pneumonia and its clinical course is also provided.

## Methods

### Design, population and organisational context

This was a prospective, descriptive and multicentre observational study of the correlation of ultrasound–DT in PC patients under telephonic follow-up for active COVID-19 infection confirmed by a diagnostic test (SARS-CoV-2 antigen test or polymerase chain reaction test). There was the clinical suspicion of pneumonia, and the use of an imaging test was indicated for its diagnosis.

The population corresponds to the VI Health Area (Vega Media del Segura) of the Region of Murcia, Spain, which covers a population of 260,820 people and is made up of 15 health centres [15]. As inclusion criteria, we considered patients of 18 and over that were clinically suspected of having COVID-19 pneumonia during follow-up by their PC physicians. These patients were subsequently referred for diagnostic confirmation by means of DT, which took place thanks to the specific high-resolution circuit between PC and the radiology service (AP-Rx). They additionally had a clinical or baseline situation that allowed the performance of LU in a sitting position, after their informed consent had been obtained. Those patients with haemodynamic instability, a previous diagnosis of pneumonia in the last 3 months or difficulty in complying with the safety standards for the prevention of COVID-19 infection were excluded.

The protocol for DT referral in our health area included those symptoms related to lung involvement: dyspnoea, tachypnoea, fever for 4 days or any symptom related to hypoxia (confusion, hypotension, cyanosis, anuria or chest pain).

In our health area, a high-resolution circuit was established between the PC and the radiology department (AP-Rx) during the first wave of the pandemic in March 2020. This circuit allows direct referral from PC in order to perform DT on patients with the clinical suspicion of COVID-19 pneumonia in less than 24 h. The patients are given appointments in a specific COVID circuit and the images obtained are immediately reported by a radiologist. If the results of the DT are normal, the patients are sent home again for PC follow-up. If, however, COVID-19 pneumonia is confirmed, they are referred to the Emergency Department (ED) [16].

This study was approved by the Hospital Morales Meseguer Clinical Research Ethics Committee (EST code: 51/20) and was carried out after informed consent had been obtained.

### Data

**Fig. 1:**
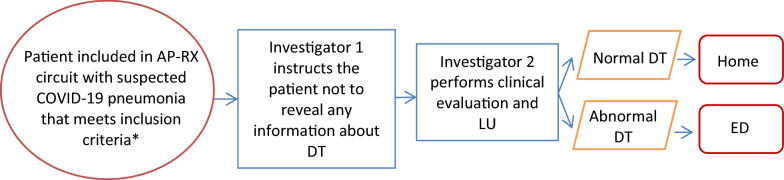
Patient flow diagram describing patient recruitment process. *Patients were recruited consecutively once Investigator 2 finished the evaluation with the previous patient. *AP-RX* specific high-resolution circuit between PC and the radiology department, *DT* digital tomosynthesis, *LU* lung ultrasound, *ED* emergency department

Data were collected by four researchers, who were 4th year resident Family and Community Medicine interns with 2 months of specific training in LU. They were organised in pairs on a 5-h working day, 2 days a week, until the entirety of the desired sample had been obtained. One of the researchers consecutively selected the patients who met the inclusion criteria after conducting the DT and then directed them to a specific room, next to the DT room, where a second researcher conducted the clinical interview and the LU (Fig. 1).

The first researcher instructed the patients not to mention any data that could reveal the result of the DT, such that the second researcher was unaware of it at all times. This, therefore, ensured that the second had no knowledge of the DT results, in order to eliminate possible bias.

**Fig. 2:**
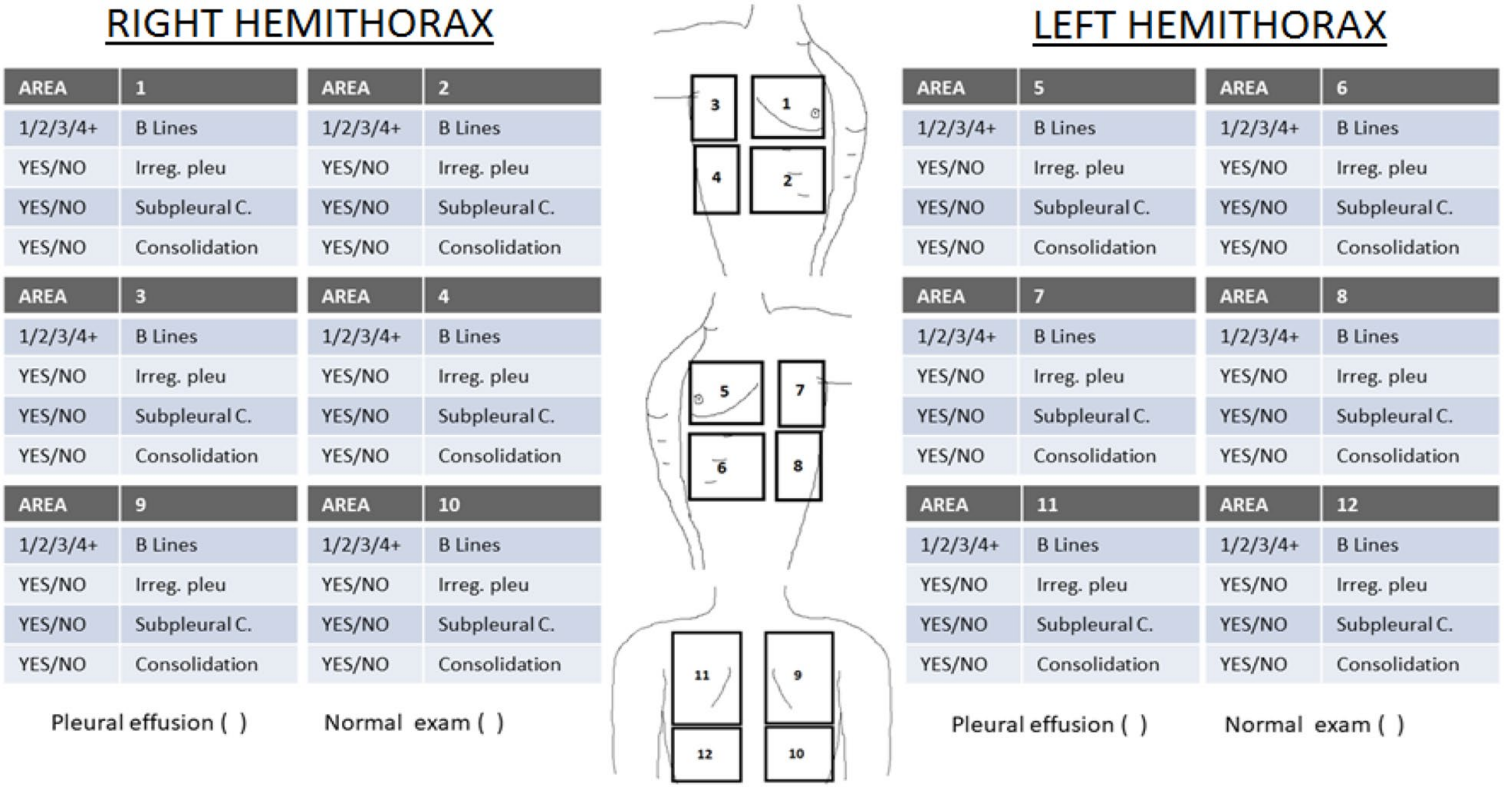
Table for ultrasound data collection. *Irreg. pleu* irregular pleural line, *Subpleural C* subpleural consolidation

The technique was performed while the patient was seated, using the SIEMENS ACUSON Freestyle™ ultrasound machine and the 3.5–5 MHz convex probe. Twelve thoracic areas were systematically evaluated, six in each hemithorax (see Fig. 2) and in a similar manner to that proposed in other studies [6, 7, 10]. The exploration technique in each field, in order from 1 to 12, consisted of a longitudinal and an oblique-transverse section, sweeping both the cranio-caudal and medio-lateral in all the intercostal spaces (the “mowing the lawn” technique).

During the LU, the researcher also employed state-of-the-art procedures [9, 17–19] to collect each patient’s demographic data, personal history, symptoms and the findings obtained from the physical and ultrasound examination. Finally, the result of the DT and the patient’s destination were noted.

The main variable employed was the number of B lines, which were categorised as follows for each lung field: “0 lines” when there was no B line, “1 B line” when there was a B line, “2 B lines” when there were two B lines, “3 B lines” when there were three B lines and “4 B lines” when four or more B lines were found or there was a confluence of them. The presence of an irregularity of the pleural line, subpleural consolidation, lung consolidation and pleural effusion was also recorded. The computerised medical history was used to verify whether the patients had been admitted, sent to the ED for a consultation, or required a new DT, as requested by their PC physician, in the following 15 days. The Brixia Index was also collected as a valid prognostic marker, together with other predictors of in-hospital mortality [20, 21]. Epidemiological data were also collected in order to contribute to a better understanding of our results.

### Statistical analysis

With regard to the statistical analysis, the *χ*^2^ statistical test was used for qualitative variables, while Fisher’s exact test was employed for samples smaller than 5 in the contingency table or < 20% as regards the expected variables. With regard to the quantitative variables, we used the Student’s *t*-test if they had a normal distribution or the Mann–Whitney *U* test otherwise (using the Kolmogorov–Smirnov test). The one-sample *t*-test was used on an individual basis to calculate the confidence interval of the means, since many authors of the literature consulted consider that 30 is a sufficient sample in order to be able to use this method [22]. When normality could not be assumed, the differences between the study groups were tested using the Mann–Whitney *U* test. A level of statistical significance of *p* < 0.05 was established.

As a complement to this analysis, a representation of the receiver operating characteristic curve (ROC) was carried out in order to discover the cut-off point in our dependent variable with a determined sensitivity (*S*), specificity (Sp), positive predictive value (PPV), negative predictive value (NPV), odds ratio (OR), positive-likelihood ratio (+LR) and negative-likelihood ratio (−LR). The IBM SPSS Statistics V.25 computer program and the R statistical program were used for this purpose. A sample size of 69 was calculated, with a significance level of 0.05 and a statistical power of 0.8.

### Inter-observer reliability

Prior to data collection, a study of reliability was conducted by the four researchers who carried out the LU. This was conducted with 16 lung clips selected by an external collaborator who was an expert in ultrasound. The degree of agreement among them was measured using Cohen’s Kappa coefficient. The Kappa coefficient was assessed by employing the Landis and Koch categorisation, 1977 (Table 1) [23]. The results obtained from the Kappa coefficient ranged between 0.45 and 1. Agreements between 0.61 and 0.80 (considerable agreement) accounted for 37.5%, and > 0.8 (almost perfect) accounted for 33.5%, signifying that 71% of the agreements were sufficiently valid to be used in the study.

**Table 1:** Cohen’s Kappa coefficient categorisation

Values of Kappa	Categorisation
0.00	No agreement
0.01–0.2	Poor agreement
0.21–0.40	Fair agreement
0.41–0.60	Moderate agreement
0.61–0.80	Substantial agreement
0.81–1.00	Almost perfect agreement

## Results

### Epidemiological context

**Fig. 3:**
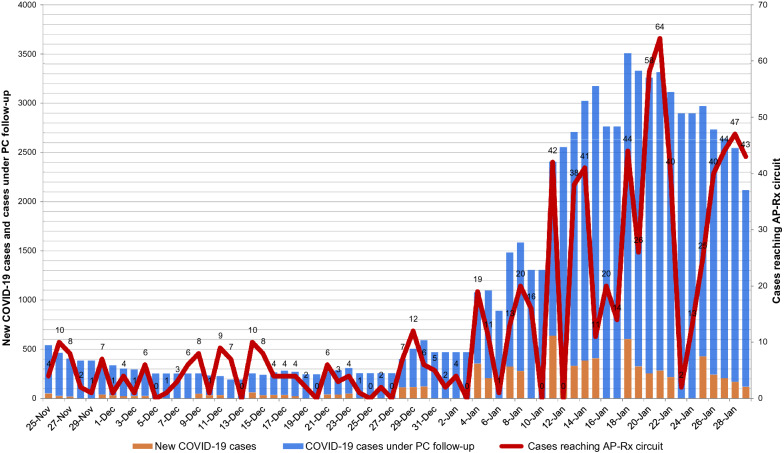
Epidemiological context

Our study period, which ranged between the 25th of November 2020 and the 29th of January 2021, included the highest 14-day cumulative number of cases per 100,000 inhabitants (from 20.3 in the period 9th to 23rd of December, to 718.12 from 7th to 21st of January), in addition to the highest number of patients on the AP-Rx circuit per day (Fig. 3).

### Clinical features

**Fig. 4:**
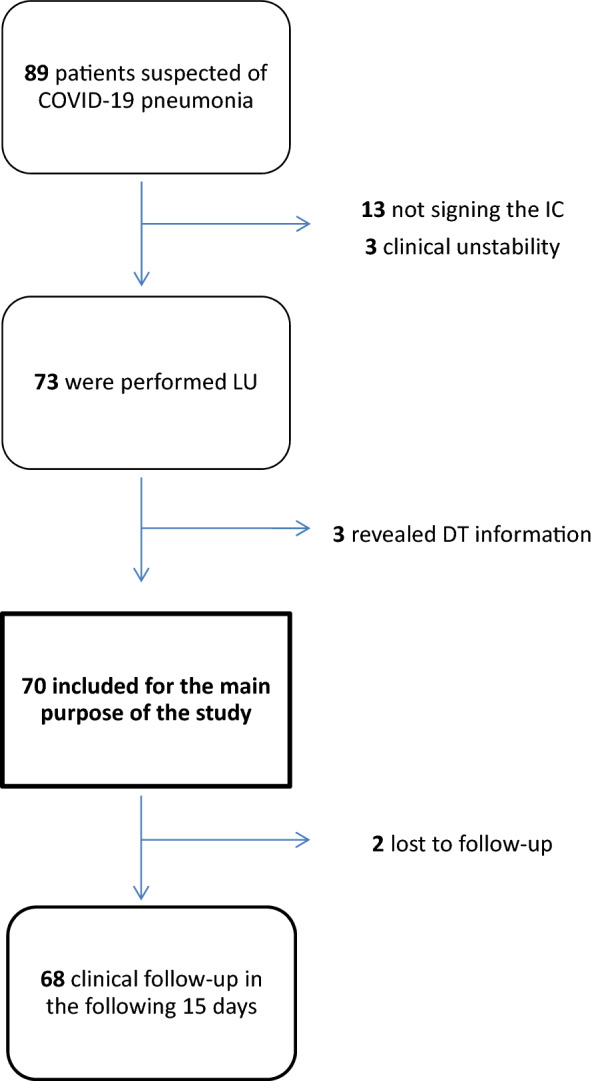
Patient flow diagram describing inclusion and exclusion criteria. *IC* informed consent, *LU* lung ultrasound, *DT* digital tomosynthesis

**Fig. 5:**
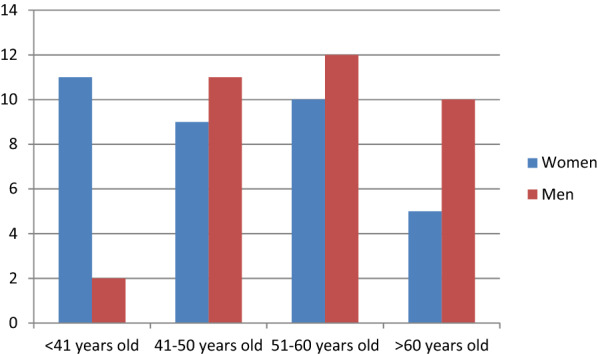
Distribution by sex and age

The initial assessment was carried out with 89 patients, 13 of whom were excluded because they had not signed the informed consent, 3 owing to clinical instability and 3 owing to the fact that the ‘blind’ nature of their DT results was compromised. This signifies that 70 patients (35 of whom were women) of between 22 and 98 years of age and from the 15 health centres in the area (100%) were eventually included in the study (Fig. 4). The distribution by sex and age, along with the clinical characteristics of the patients, is shown in Table 2 and Fig. 5.

**Table 2:** Patient distribution by sex and clinical characteristics

Variable	*n* (%)	*n* (%)	Total	Differences between men and women
Sex	Men *n* = 35 (50%)	Women *n* = 35 (50%)	70	
Age	56.11 (51.84–60.39)	48.2 (43.50–52.90)	52.16	*p* = 0.014
Pneumonia	24 (68%)	16 (45.7%)	40 (57.14%)	NS
Arterial hypertension	13 (37.1%)	7 (20%)	20 (28.6%)	NS
Diabetes mellitus	6 (17.1%)	2 (5.7%)	8 (11.4%)	NS
Dyslipidaemia	8 (22.9%)	4 (11.4%)	12 (17.1%)	NS
Smoker	9 (25.7%)	4 (11.4%)	13 (18.6%)	NS
COPD/asthma	7 (20.0%)	8 (22.9%)	15 (21.4%)	NS
Cardiopathy/chronic heart failure	5 (14.3%)	2 (5.7%)	7 (10%)	NS
Days from the onset of symptoms	8.57	10.06	9.31	NS
Fever	22 (62.9%)	18 (51.4%)	40 (57.1%)	NS
Dyspnoea	19 (54.3%)	20 (57.1%)	39 (55.7%)	NS
Cough	26 (74.3%)	27 (77.1%)	53 (75.7%)	NS
Expectoration	8 (22.9%)	6 (17.1%)	14 (20%)	NS
Pathological lung auscultation	11 (31.4%)	9 (25.7%)	20 (28.6%)	NS
Oxygen saturation	96.10 (95.2–97.01)	97.24 (96.78–97.8)	97.17	*p* = 0.034
Mean time of evaluation (min)	15.90	16.79	16.33	NS

*COPD* chronic obstructive pulmonary disease, *NS* not significant, *min* minutes

A total number of 40 patients were diagnosed as having pneumonia by means of DT (57.14%). The patients with pneumonia were older, had a higher proportion of arterial hypertension and a lower sO_2_ (Table 3). There was a higher non-significant proportion of men (60%) with an OR of 1.58 (*p* = 0.053). Patients over 55 had pneumonia with an OR of 2.75 (*p* = 0.049). Only one patient (1.4%) had a diagnosis additional to that of pneumonia (lung granuloma).

**Table 3:** Patients’ clinical distribution by the presence of pneumonia

	Pneumonia (*n* = 40)	No pneumonia (*n* = 30)	
Age	55.7	47.4	*p* = 0.011
Sex			
Women	16	19	NS
Men	24	11	
Arterial hypertension	17	3	*p* < 0.003
Diabetes mellitus	7	1	NS
Dyslipidaemia	10	2	NS
Smoker	7	6	NS
COPD/asthma	9	6	NS
Cardiopathy/chronic heart failure	5	2	NS
Days from the onset of symptoms	8.85	9.93	NS
Fever	24	16	NS
Dyspnoea	24	15	NS
Cough	33	20	NS
Expectoration	10	4	NS
Pathological lung auscultation	14	6	NS
Oxygen saturation	96.11	97.58	*p* < 0.05
Mean time of evaluation (min)	16.91	15.4	NS
Brixia Index	5.26	0	*p* < 0.001

*COPD* chronic obstructive pulmonary disease, *NS* not significant, *min* minutes

### Ultrasound correlation and COVID-19 pneumonia

**Fig. 6:**
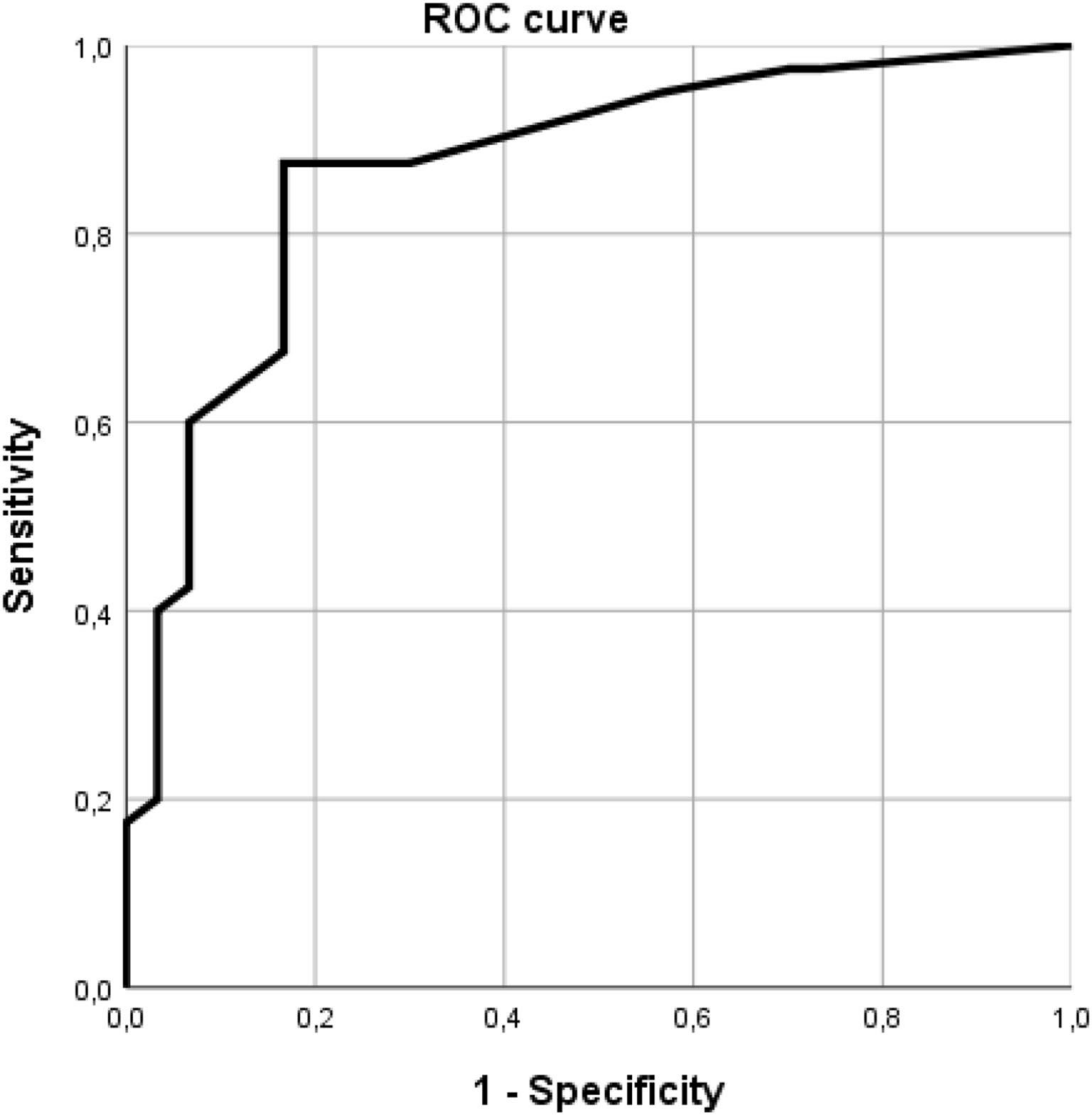
ROC curve of the sum of the B lines of all the lung fields and pneumonia in digital tomosynthesis. *ROC* receiver operating characteristic curve

The mean sum of the B lines of all the fields of those patients who were diagnosed as having pneumonia by means of DT (16.53, 95% CI 13.23–19.81) was significantly higher than those who did not have pneumonia (4.3, 95% CI 2.04–6), with *p* < 0.001. The area under the curve (AUC) was 0.87 (95% CI 0.78–0.96, *p* < 0.001), and when establishing a cut-off point of six B lines or more, the *S* was 0.875 (95% CI 0.77–0.98, *p* < 0.05), the Sp was 0.833 (95% CI 0.692–0.975, *p* < 0.05), the PPV was 0.875 (95% CI 0.77–0.98, *p* < 0.05) and the NPV was 0.833 (95% CI 0.692–0.975, *p* < 0.05) for the diagnosis of COVID pneumonia (Table 4 and Fig. 6). The +LR was 5.25 (95% CI 2.34–11.79) and the −LR (−LR) was 0.15 (95% CI 0.07–0.34). No significant differences between men and women or age groups were found in the subgroup analysis.

**Table 4:** Main ultrasound findings according to the presence of pneumonia in DT

Ultrasound findings	Pneumonia (%) [*S*]	No pneumonia (%) [Sp]	OR, +LR, −LR
≥ 6 B lines, adding all fields, maximum 4 B lines per lung field	35/40 (87.5%) [*S* = 0.875]	5/30 (16.6%) [Sp = 0.833]	OR = 35 *p* < 0.001 +LR = 5.25 (95% CI 2.34–11.79) −LR = 0.15 (95% CI 0.07–0.34)
≥ 4 B lines or confluent B lines at least in one lung field	29/40 (72.5%) [*S* = 0.725]	5/30 (16.7%) [Sp = 0.833]	OR = 13.18 *p* < 0.001 +LR = 4.35 (95% CI 1.91–9.90) −LR = 0.33 (95% CI 0.19–0.56)
≥ 3 B lines or confluent B lines in at least one lung field	34/40 (85%) [*S* = 0.85]	11/30 (36.7%) [Sp = 0.63]	OR = 9.78 *p* < 0.001 +LR = 2.32 (95% CI 1.42–3.78) −LR = 0.24 (95% CI 0.11–0.52)
Irregular pleural line in at least 2 lung fields in absence of subpleural consolidation	13/29 (44.8%) [*S* = 0.448]	8/30 (26.7%) [*E* = 0.733]	OR = 2.23 *p* < 0.05 +LR = 1.68 (95% CI 0.82–3.45) −LR = 0.75 (95% CI 0.51–1.11)
Subpleural consolidation	11/40 (27.5%) [*S* = 0.275]	0/30 (0%)	+LR: NC −LR: 0.72 (95% CI 0.60–0.89)
Condensación	0/40	0/30	NC
Pleural effusion	6/40 (15%)	2/30 (6.7%)	NS

DT: digital tomosynthesis; *S*: sensitivity; Sp: specificity; OR: odds ratio; +LR: positive-likelihood ratio; −LR: negative-likelihood ratio; CI: confidence interval; NC: not calculable; NS: not significant

Of the 40 patients with pneumonia (27.5%), 11 had no field with four or more B lines and 29 (72.5%) had at least one. Of the 30 patients without pneumonia (16.7%), 5 had a field with four or more B lines and 25 (83.3%) did not have any fields with four or more B lines. The main ultrasound findings according to the presence of pneumonia are shown in Table 4.

There were 11 patients with at least one subpleural consolidation in one field, and pneumonia was found in all of them when employing DT. With regard to the irregularity of the pleural line, when selecting only those patients without subpleural consolidation, there was statistical significance as regards the number of fields with an irregular pleural line variable (normal vs. pneumonia, 0.7 vs. 1.83 fields, *p* = 0.015), with AUC = 0.653 *p* = 0.044 and with an *S* = 0.24 and Sp = 1 with a cut-off point in two or more affected fields.

The mean Brixia score for pneumonia was 5.26 out of 18 points (with a range of 2 to 11 points), with no differences between men and women. The mean time required to perform LU was 16.33 min, with no significant differences between sex and the presence of pneumonia.

### Correlation with prognosis

No patient with normal DT results required admission or a new consultation in the following 15 days. Of the patients with pneumonia, those who required admission (*n* = 7) had a greater number of B lines than those who did not (25.7 vs. 14.57, *p* = 0.008). There was one patient with seven B lines and pneumonia who was admitted for a reason other than pneumonia (lung granuloma). No patient with five B lines or fewer was admitted. Of those who were not admitted (*n* = 63), eight were sent to the ED for consultations in the following 15 days (12.7%) (it was not possible to follow up two patients), and there were no significant differences in the number of B lines of those who had consultations and those who did not (14.6 vs. 9.05 *p* = 0.16). Only two patients with five or fewer B lines (6, 7%) went to the emergency department for consultations in the following 15 days and there was no need for additional treatment. Furthermore, there were six patients with 6 or more B lines (18, 2%) who had consultations in the following 15 days, all of whom had 16 or more B lines.

An age equal to or greater than 55 was independently associated with hospital admission, with OR = 9.00 (*p* = 0.014). No other clinical or ultrasound parameter was associated with admission.

The presence of subpleural consolidation was not associated with admission, but was associated with being sent for a consultation in the ED in the following 15 days (OR = 10, *p* = 0.025). Of the patients who were discharged with a subpleural consolidation, 37.5% had consultations in the following 15 days, compared to 6% who were discharged without a subpleural consolidation. Neither the sum of B lines nor any other ultrasound or clinical parameter was associated with consultation in the following 15 days.

Overall, with regard to patients under 55 with sO_2_ > 95% and without a subpleural consolidation, the number of B lines had an AUC of 0.972 (95% CI 0.919–1.000) as regards the need for admission, while with a cut-off point of 21 B lines, the *S* was 1 and the *E* was 0.97. In other words, in this subset of patients, the presence of < 21 B lines (a maximum of 4 per field) ruled out the need for admission. Only 2 out of 37 patients with these characteristics in our cohort required admission, and had 28 and 29 B lines, respectively.

The patients who required admission (*n* = 7) met at least one of the following criteria: 55 years old or over, sO_2_ ≤ 95% and the presence of at least one subpleural consolidation or ≥ 21 B lines.

## Discussion

The presence of lung involvement in a patient with COVID-19 may be a reason for hospital referral or close follow-up in PC [9]. Its confirmation could, therefore, help physicians during the decision-making process.

This study verifies the good sensitivity and specificity of LU in PC when performed by family physicians with specific training, with results similar to those obtained in studies that have, to date, included patients only in the hospital or residential setting [11] and for whom LU has a considerable inter-observer agreement similar to that attained in other studies [24].

DT rather than CT has been used as a reference test owing to the lower availability of the latter and because its choice is not feasible owing to radiation and access limitation [3]. Moreover, the fact that DT is performed in a specific AP-Rx circuit [16] improves the precision of chest radiography for subtle opacities [4, 5], thus making it appropriate for our population in PC, since it is a population with a milder disease. The fact that our population has a milder disease can be confirmed in our study by taking into account variables such as the Brixia Index score, which was low (an average of 5.26 out of 18 points) and a lower number of admissions into the COVID-19 unit and ICU than in other studies (10% and 0% vs. 16% and 10%) [10]. We, therefore, consider that the reference test is valid.

We found that the LU performed best as regards diagnosing COVID-19 pneumonia when a cut-off point was established at six B lines, counting a maximum of four B lines per field and adding all the fields. If we compare this with considering only four or more B lines or a confluence of B lines in at least one field or 3 or more B lines or a confluence in at least one field, the *S* and Sp are lower (see Table 4). It would, therefore, appear reasonable to assume that the performance of LU in the diagnosis of COVID-19 pneumonia improves when evaluating all lung fields and considering the largest number of artefacts.

We found that the *S*, Sp and AUC (0.875, 0.833 and 0.87) were similar to those attained in other studies in the hospital setting (*S* 0.68–1 and *E* 0.546–0.89 and AUC 0.745–0.866), as were the PPV and NPV (in our study, these were 0.875 and 0.833, when compared to the 0.54–0.92 and 0.36–0.98 attained in other studies) [1, 6, 10, 11, 17, 18, 24, 25]. It is necessary to bear in mind that in our study, although there were no differences between the age groups in terms of AUC, there appears to be a trend towards a higher *S* and a lower Sp (1 and 0.676) in older patients (> 60). This observation coincides with that of another study in nursing homes that found an Sp of 0.48 [26].

Both the number of B lines and the presence of a subpleural consolidation could similarly be correlated with a poor prognosis (more admissions and consultations in the following 15 days, respectively), as found in other studies carried out in PC [9] and in the ED field [10]. Furthermore, the presence of five or fewer B lines rules out the need for admission, although further research is required in order to confirm this. What it is possible to state is that LU could be a valid tool in PC for decision-making and the optimisation of resources, together with the assessment of pulse oximetry and the age of the patient.

The study was developed during the so-called “third wave” of the COVID-19 pandemic. All the patients included had obtained a positive microbiological diagnostic test for coronavirus, and the results should be understood in that context. The external validity of this study is based precisely on the fact that the operators are PC physicians who know the patients’ symptoms and the microbiological test and perform the LU, similar to that which occurs in clinical practice in PC.

The limitations of this study include the following. First, although DT is a promising diagnostic tool [4, 5], it has yet to be well studied in the context of COVID-19 pneumonia. Second, as any patient with COVID-19 infection may have lung involvement, it is necessary to take into account that this study has been carried out only with patients with specific symptoms suggesting the need for hospital management. Another important limitation is that immobilised or institutionalised patients were not included, signifying that other studies would be necessary in order to corroborate validity as regards these PC patients.

**Fig. 7:**
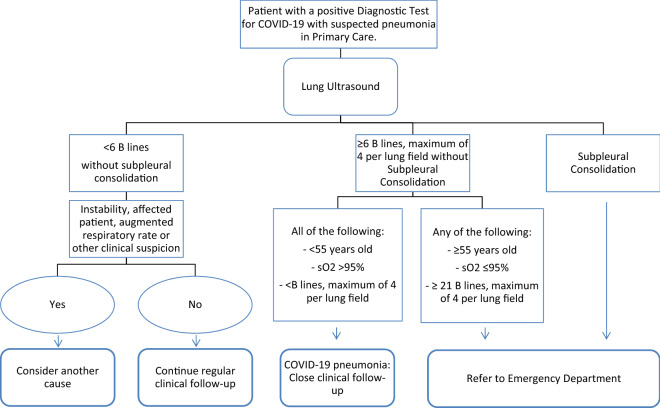
Management proposal for patients with suspected COVID-19 pneumonia in PC

Bearing our results in mind, once COVID-19 pneumonia is suspected, the most important clinical variables for the diagnosis of or the need for admission are age, oxygen saturation and lung ultrasound. We, therefore, propose the following management algorithm for suspected COVID-19 pneumonia in PC (Fig. 7).

## Conclusions

Point-of-care ultrasound in primary care has good sensitivity and specificity when compared to digital tomosynthesis as regards the diagnosis of COVID-19 pneumonia in patients with the clinical suspicion of pulmonary involvement. In the case of these patients in particular, clinical ultrasound findings could, along with age and oxygen saturation, improve decision-making in primary care. We propose a management algorithm for these patients, and as future work, there is a need to confirm its usefulness and as regards developing statistical models with which to diagnose COVID-19 pneumonia and predict its severity. Further research is particularly required for elderly and immobilised patients in the primary care setting.

## Data Availability

The datasets used and/or analysed during the current study are available from the corresponding author on reasonable request.

## Abbreviations

+LR: Positive-likelihood ratio
−LR: Negative-likelihood ratio
AP-Rx: Specific high-resolution circuit between primary care and the radiology department
AUC: Area under the curve
CI: Confidence interval
COVID-19: Coronavirus disease 19
CT: Computed tomography
DT: Digital tomosynthesis
ED: Emergency department
LU: Lung ultrasound
NPV: Negative predictive value
OR: Odds ratio
PC: Primary care
PPV: Positive predictive value
ROC: Receiver operating characteristic
*S*: Sensitivity
Sp: Specificity
sO_2_: Oxygen saturation
